# Knowledge, attitudes and beliefs on contributing factors among low back pain patients attending outpatient physiotherapy treatment in Malawi

**DOI:** 10.4102/sajp.v73i1.395

**Published:** 2017-10-31

**Authors:** Nesto Tarimo, Ina Diener

**Affiliations:** 1 Department of Physiotherapy, College of Medicine, University of Malawi, Malawi; 2 Department of Physiotherapy, University of the Western Cape, South Africa

## Abstract

**Background:**

Low back pain (LBP) affects many people globally. Its aetiology is not clear. Patients lack knowledge of its contributing factors and have negative perception about their LBP. This study aimed to identify knowledge, attitudes and beliefs regarding the perceived contributing factors to LBP among patients attending physiotherapy outpatient departments in Malawi. This information can possibly facilitate planning of a LBP education programme in Malawi.

**Methods:**

A quantitative cross-sectional survey was conducted, using a six-part self-administered questionnaire with questions on demographic information, participants’ attitudes and beliefs regarding their LBP, knowledge about the course and causes of LBP, beliefs regarding nine contributing factors to LBP (identified in a Delphi study) and the sources of the participants’ knowledge. Data were analysed descriptively using the Statistical Package for Social Sciences (version 19.0). A Chi-square test was used to determine any association between variables (alpha 0.05). All ethical procedures were strictly followed.

**Results:**

Most participants (186, 91.2 %) did not manage to answer all six questions regarding knowledge correctly and were regarded as ‘partially knowledgeable’ about the course and causes of LBP. More than half (67%) portrayed negative attitudes and beliefs about LBP in general. The findings also showed a statistically significant relationship between knowledge, attitudes and beliefs (*p* = 0.04).

**Conclusion:**

This study highlighted that many patients with LBP in Malawi are not adequately knowledgeable about LBP and hold negative attitudes and beliefs regarding their LBP. Therefore, LBP management programmes in Malawi should include education programmes aimed at empowering patients with knowledge regarding LBP, as well as changing their negative attitudes and beliefs about their pain. Patients’ understanding of the cause and nature of their pain may enhance the achievement of treatment goals.

## Introduction

Low back pain (LBP) has been reported as a worldwide health problem, affecting individuals physically, socio-economically and psychologically (Hoy et al. 2012; Manchikanti et al. 2014). Although most epidemiological studies on the prevalence of LBP have been conducted in developed countries (Bruce et al. 2004; Ghaffari et al. 2006), there is not much difference between the prevalence of LBP in developed and developing countries (Louw, Morris & Grimmer 2007). In developed countries, the lifetime prevalence is between 60% and 70% (WHO 2004). In Africa alone, it ranges between 28% and 74% and is most likely to increase globally in the next few years (Hoy et al. 2012; Louw et al. 2007).

The exact causes of LBP are often difficult to identify, and both clinicians and patients are left with uncertainties, leading to varied broad practices in the choice of management of LBP (Adam 2009; Cole & Grimshaw 2003). In addition, most patients living with LBP lack knowledge regarding causes and contributing factors of LBP (Ng’uurah & Frantz 2006). This is despite various treatment guidelines for LBP proposing that besides physical treatment and exercises, advice and health education should be part of the treatment plan (Koes, Van Tulder & Thomas 2006). Health education will not only enhance peoples’ knowledge about pain, but might also change their negative attitudes and beliefs regarding their pain, and thereby promote the achievement of the desired clinical outcomes (Henrotin et al. 2006). This in turn may decrease the number of patients living with acute LBP and transitioning to living with chronic LBP (Fowler & Dabco 2004). More recently, pain neuroscience education has demonstrated good results in the management of LBP populations, changing pain cognitions and improving activity performance among patients (Clarke, Ryan & Martin 2011; Louw et al. 2011; Ryan et al. 2010).

The general public, including patients living with LBP, lack knowledge about the causes and contributing factors of LBP (Allock, Elkan & Williams 2007; Ng’uurah & Frantz 2006; Tavafian et al. 2004). This implies that during management of LBP, patients’ knowledge, attitudes and beliefs about their pain should be identified and followed by education regarding their pain. In Malawi, LBP is one of the leading musculoskeletal conditions treated at hospital physiotherapy outpatients’ departments. However, no information is available regarding patients’ knowledge, attitudes and beliefs regarding their LBP. This study thus aimed to establish the knowledge (understanding), attitudes and beliefs regarding perceived contributing factors to LBP among patients seeking physiotherapy management for their LBP at health centres in Malawi.

## Methods and materials

This study was conducted in the physiotherapy outpatient departments of Queen Elizabeth and Kamuzu Central hospitals in Malawi. A quantitative research design using a cross-sectional survey was used. Convenience sampling was used as the recruitment method with a self-administered questionnaire for data collection. The questionnaire consisted of six sections. In the first two sections, demographic and social data as well as information on the current state of participants’ LBP was gathered. The third section, on the attitudes and beliefs regarding LBP, was based on the Back Beliefs Questionnaire (Symonds et al. 1996) and the Survey of Pain Attitude Questionnaire (Jensen et al. 1994). The fourth section, based on pertinent literature and the LBP Knowledge Questionnaire (Maciel et al. 2009), sought participants’ knowledge and understanding of the course and causes of LBP in general. The fifth section of the questionnaire explored participants’ beliefs regarding nine contributing factors to LBP. Firstly, a literature review was conducted to identify the contributing factors to LBP. Fifteen contributing factors were identified (Table 1). A Delphi method was then used to establish the most important contributing factors to LBP by seeking the opinion from a panel of experts (physiotherapists with more than 2 years of experience in the field of LBP). Twenty experts were identified and 15 agreed to participate. The Delphi method included three rounds (Custer, Scarcella & Stewart 1999). In the first round, the 15 contributing factors were sent to the experts and they were requested to add any other contributing factors. After the first round, a new list of 38 factors was compiled, which included the factors added by the experts (Table 2). In the second and third rounds, the experts were asked to rate the 38 factors on a four-point Likert scale ranging from 1 = ‘not important’ to 4 = ‘very important’. Seventy per cent or higher agreement on an element was interpreted as an acceptable level of consensus (Hsu & Standford 2007). Factors rating 30% or less were eliminated from the list. All factors (nine in total) that were ranked 70% and above were identified and included in the questionnaire (Table 3). The last section of the questionnaire identified the sources of the participants’ knowledge and their views on their own LBP. The developed questionnaire was tested in a pilot study to determine the user-friendliness, the clarity of the instrument as well as time to complete the questionnaire (De Vos 2002). Ten patients with LBP participated in the pilot study and they were excluded from the main study. The pilot study revealed that a few questions were slightly unclear to the participants, because of the use of medical terminologies. The questions were revised and the modifications to those medical terms were made by replacing them with simple terms (Table 4).

**TABLE 1:** List of causing or contributing factors to low back pain from a review of the literature.

No.	Contributing factors
1.	Analgesic dependency
2.	Anxiety
3.	Depression
4.	Fear avoidance beliefs (Fear that movement may injure structures in the back)
5.	Flexion combined with compressive force to the lumbar spine (e.g. in lifting heavy objects)
6.	Frequency of twisting and bending of the spine during work of sport activities
7.	Obesity
8.	Physically demanding work (as perceived by the patient)
9.	Poor or unhappy social environment at work
10.	Prolonged sitting (more than 30 min)
11.	Repetitive heavy lifting
12.	Smoking
13.	Social psychological stress and events in life
14.	Stressful life events (e.g. caring for the sick or facing death)
15.	Whole-body vibration (e.g. in truck driving and other work activities)

*Source*: Authors’ own work

**TABLE 2:** List of causing or contributing factors to low back pain after round 1 of the Delphi study.

No.	Contributing factors
1.	Age
2.	Analgesic dependency
3.	Anxiety
4.	Catastrophising
5.	Co-morbid diseases (e.g. diabetes, hypertension, cardiac pathology)
6.	Compensation situations (e.g. work injuries, third part)
7.	Congenital malformations (e.g. loss of lumbar curvature)
8.	Degenerative joint disease from old age
9.	Depression
10.	Fear avoidance beliefs (fear that movement may injure structures in the back)
11.	Flexion combined with compressive force to the lumbar spine (e.g. in lifting heavy objects)
12.	Frequency of twisting and bending of the spine during work of sport activities
13.	Gender
14.	Leg length discrepancy
15.	Obesity
16.	Passive coping
17.	Patient’s lack of understanding of pathology
18.	Perceived future problems
19.	Perception on workload
20.	Physically demanding work, as perceived by the patient
21.	Poor mattress quality
22.	Poor or unhappy social environment at work
23.	Posture
24.	Pregnancy
25.	Previous epidural anaesthetic spinal block
26.	History of back pain
27.	Prolonged sitting (more than 30 min)
28.	Repetitive heavy lifting
29.	Self-efficacy beliefs
30.	Smoking
31.	Social psychological stress and events in life
32.	Some sport activities (e.g. skiing )
33.	Spouse relations – a solicitous spouse may increase pain behaviour
34.	Stressful life events (e.g. caring for the sick or facing death)
35.	Trauma or injury
36.	Trigger points in gluteus muscles
37.	Types of the chair used at home or work
38.	Whole-body vibration (e.g. in truck driving and other work activities)

*Source*: Authors’ own work

**TABLE 3:** Highest ranked contributing factors to low back pain (Delphi study, *n* = 15).

Contributing factor	Frequency	
	*n*	%
1. Compensation situations (e.g. injuries at work places)	11	73.3
2. Physically demanding works	12	80.0
3. Trauma or injury at the back	12	80.0
4. Whole-body vibration	12	80.0
5. Fear avoidance beliefs	13	86.7
6. History of back pain	13	86.7
7. Twisting and bending of the spine	13	86.7
8. Flexion combined with compressive forces	14	93.3
9. Repetitive heavy lifting	14	93.3

*Source*: Authors’ own work

**TABLE 4:** The simplified terms from the Pilot study.

Section in the questionnaire	Question No.	Term	Changed to
A-2	1	Episodic	Intermittent: comes and goes
B-2	2(b)	Athrosis	Joint problems
	2(b)	Herniated disc	Slipped disc

*Source*: Authors’ own work

### Ethical consideration

Ethical clearance was obtained from the University of the Western Cape and the College of Medicine in Malawi with certificate numbers 10/9/22 and P.10/10/1005, respectively. Participants were informed regarding the study before participation and all participants signed informed consent.

## Results

### Socio demographic characteristics

Two hundred and five participants were recruited, of whom 109 (53%) were women, with a mean age of 47.74 (± 13.29) years. Table 5 illustrates the socio demographic characteristics of the study sample.

**TABLE 5:** Socio demographic characteristics of the study sample (*n* = 205).

Variable	Frequency (*n*)	(%)
Gender		
Males	96	46.8
Females	109	53.2
Age group		
15–24 years	8	3.9
25–34 years	27	13.2
35–44 years	44	21.5
45–54 years	62	30.2
55–64 years	43	21.0
≥ 65 years	21	10.2
Level of education		
Never attended school	66	32.0
Primary level	98	47.8
Secondary level	34	16.6
Tertiary level	7	3.4
Marital status		
Single	48	23.4
Married	104	50.7
Divorced	19	9.3
Separated	3	1.5
Widow	25	12.2
Widower	6	2.9
Residential area		
Rural	47	22.9
Urban	158	77.1

*Source*: Authors’ own work

Mean age 47.74 years (SD = 13.29).

### Low back pain frequency and duration

The majority of the participants 99 (48.3%) reported that the pain was continuous, while 81 participants (39.5%) indicated that the LBP was episodic, and only 25 participants (12.2 %) reported a first-time (or once-off) episode of LBP. Seventy-eight per cent of all participants reported LBP for more than 6 months, followed by 21 (10.2%) who reported LBP for 2–3 months and 24 (11.8%) reported pain for 1 month or less.

### Attitudes and beliefs about their own low back pain

Twelve statements were provided in the questionnaire (Table 6), requiring participants to indicate from strongly disagree to strongly agree, their opinion regarding their own LBP. The majority of the participants, 190 (93%), reported fear of movement and activity avoidance because of their LBP, while 147 (72%) believed that their LBP eventually would prevent them from working and would remain with them for the rest of their lives. Thirty-three per cent of all participants demonstrated positive attitudes and beliefs on all statements but the majority of participants, 137 (66.8%), demonstrated negative attitudes and beliefs regarding their LBP.

**TABLE 6:** Summary of responses of participants on attitudes and beliefs regarding their own low back pain.

Statements	Agree	Do not know	Disagree
1. People with LBP should avoid movement as it may cause more injury	**190 (92.7%)**	5 (2.4%)	10 (4.9%)
2. Pain acceptance facilitates recovery from LBP	111 (54.1%)	36 (17.6%)	58 (28.3%)
3. Only health personnel can cure LBP	**175 (85.4%)**	9 (4.4%)	21 (10.2%)
4. Self-management on your LBP has no effect on recovery	82 (40.0%)	49 (23.9%)	74 (36.1%)
5. LBP will eventually stop you from working	**147 (71.7%)**	22 (10.7%)	36 (17.6%)
6. Your LBP will last with you for the rest of your life	26 (12.7%)	83 (40.5%)	**96 (46.8%)**
7. LBP will never stop you doing what you really want to do	85 (41.5%0	28 (13.7%)	**92 (44.8%)**
8. Because of your LBP, abstain from your duties and avoid physical activity	**140 (68.3%)**	14 (6.8%)	51 (24.9%)
9. Having LBP may mean you will end up with disability	55 (26.8%)	53 (25.9%)	**97 (47.3%)**
10. You can control the amount of pain you feel by changing your thoughts	64 (31.2%)	66 (32.2%)	75 (36.6%)
11. To know more about your pain, the best way is to go to the healthcare facility	**191 (93.2%)**	6 (2.9%)	8 (3.9%)
12. LBP gets progressively worse later in life	**143 (69.8%)**	39 (19.0%)	23 (11.2%)

*Source*: Authors’ own work

For clarity, the responses ‘strongly agree’ and ‘agree’ were collapsed into ‘agree’ and the responses ‘strongly disagree’ and ‘disagree’ were collapsed into ‘disagree’.

LBP, low back pain.

### Knowledge of the participants about the course and causes of low back pain

Participants were given a series of six questions to indicate their choices on the factors that possibly cause LBP (Table 7). The majority of the participants, 186 (91.2%), were partially knowledgeable on the course and causes of LBP in general. Only 18 (8.8%) of them answered all questions correctly and were considered to be fully knowledgeable on the course and causes of LBP. Table 8 summarises the participants’ knowledge on causes and contributing risk factors to LBP.

**TABLE 7:** Participants’ agreement on individual statements regarding the course and causes of low back pain.

Statement	Agreed		Opinion and results from the literature
	(*n*)	(%)	
**Q 1.** With regard to acute LBP, mark the two most correct statements			
**a. The great majority of patients recover in 3 weeks**	6	2.9^a^	**Yes** (Pengel et al. 2003)
b. After recovery and improvement of pain the patient	29	14.1	**No** (Cassidy et al. 2005; Pengel et al. 2003;)
c. Instructions on protection of spine are only important during crisis	17	84.4	**No** (Waddell, Feder & Lewis 1997)
**d. The actions and exercises for spine protection and energy conservation are a routine in a patient with history of LBP because relapses are frequent**	182	88.8^a^	**Yes** (Maciel et al. 2009; Samanta & Beardsley 1999)
**e. I don’t know**	**10**	**4.9**	
**Q 2. These could cause LBP: Mark the two most correct statements**			
**a. Previous history of LBP and physically demanding work**	51	24.9^a^	**Yes** (Kerr et al. 2001)
**b. Postural problems, joint problems and slipped disc**	172	83.9	**Yes** (Louma et al. 2000; Vindigni et al. 2005) (Supported by 87% of Delphi experts)
c. **Trauma and injuries to the back**	161	78.5^a^	
d. Diabetes	5	2.4	**No** (No indication in the literature)
e. I don’t know	10	4.9	
**Q 3**. These factors could contribute to development of LBP (**You can indicate more than one**)			
**a. Physical factors**	111	54.1^a^	**Yes** (Soucy, Truchon & Cote 2006)
**b. Social factors**	30	14.6^a^	**Yes** (Yip & Ho 2001)
**c. Psychological factors**	16	7.8^a^	**Yes** (Heymans et al. 2010)
**d. Work-related factors**	112	54.6^a^	**Yes** (Heymans et al. 2010)
e. None of the above	14	6.8	
f. I don’t know	46	22.4	
**Q 4**. To protect your spine: Mark **two** most correct statements (i.e. which two are most likely to protect your spine?)			
a. The best way to sleep is on your stomach	68	33.2	**No** (Pithwa 2011)
**b. Sit down to put on your socks and shoes**	88	42.9^a^	**Yes** (Maciel et al. 2009)
c. Pick up objects from the floor without bending the knees	51	24.9	**No** (Pithwa 2011)
**d. Wash the dishes with stomach leaned against the sink**	26	12.7^a^	**Yes** (Maciel et al. 2009)
e. I don’t know	87	42.4	
**Q 5.** Again in relation to spinal protection; Mark **one** statement which is **wrong** (i.e. which one **cannot** protect your spine?)			
a. Get out of the bed carefully, turning sideways with the help of your hands	6	2.9	**Yes** (Adams 2004)
b. Avoid carrying too much weight on one side of your body (divide the load between both arms)	6	2.9	**Yes** (Smedley et al. 1995)
c. Avoid twisting your spine	6	2.9	**Yes** (Okunribido, Magnusson & Pope 2008)
d. **Wear high heel shoes everyday**	183	89.3	**No** (Lee, Jeong & Freivalds 2001**)**
e. I don’t know	4	2.0	
**Q 6.** The following could be reasons for LBP (**You can indicate more than one**)			
a. Poor sitting	116	56.6^a^	**Yes** (Samad et al. 2010)
b. Bending and twisting the trunk	122	59.5^a^	**Yes** (Onkunribido et al. 2008)
c. Poor lifting of heavy loads	187	91.2^a^	**Y**es (Foppar & Novack 1996)
d. Poor working environment	113	55.1^a^	**Yes** (Kerr et al. 2001; Vindigni et al. 2005)
e. Physical inactivity	27	13.2^a^	
f. I don’t know	2	1.0	
g. Others^b^	13	6.3	

*Source*: Authors’ own work

LBP, low back pain.

a , The correct statement(s) based on the literature.

b , Includes mismanagement during child birth, climbing mountain, prolonged standing and poor mattress.

**TABLE 8:** Summary of the participants’ knowledge on the course and causes of low back pain.

Portrayed knowledge per individual question	Frequency	
	(*n*)	(%)
**Q 1. Choices of the participants with regards to acute LBP**		
All chosen statements are wrong	22	10.7
Only one correct statement chosen	178	86.8
Both the statements correctly chosen	5	2.4
**Q 2. Choices of the participants with regard to causes of LBP**		
All chosen statements are wrong	14	6.8
Only one correct statement chosen	171	83.4
Both the correct statements chosen	20	9.8
**Q 3. Choices of the participants with regard to factors contributing to LBP**		
All chosen statements are wrong	60	29.3
Only one correct statement chosen	45	22.0
Only two correct statements chosen	83	**40.5**
Only three correct statements chosen	12	5.9
All four statements correctly chosen	5	2.4
**Q 4. Choices of the participants with regard to spine protection**		
All chosen statements are wrong	106	51.7
Only one correct statement chosen	83	40.5
Both the statements correctly chosen	16	7.8
**Q 5. Choices of the participants in relation to spine protection again**		
Wrong statements	22	10.7
Correct statement	183	89.3
**Q 6. Choices of the participants on the possible reasons for LBP**		
All chosen statements are wrong	2	1.0
Only one correct statement chosen	4	2.0
Only two correct statements chosen	85	41.5
Only three correct statements chosen	60	29.3
Only four correct statements chosen	47	22.9
All five correct statements correctly chosen	7	3.4

*Source*: Authors’ own work

LBP, low back pain.

### Attitudes and beliefs of contributing factors to their low back pain

The minimum level of consensus during the Delphi study was set at 70%. Nine contributing factors were ranked high by the experts and therefore included in the questionnaire of this study. Patients living with LBP were then requested to indicate their opinions regarding these nine factors, with the responses ranging from strongly disagree to strongly agree. The majority (86.3%) of the participants living with LBP agreed that all nine factors could contribute to the development and/or worsening of their LBP.

### Sources of participants’ knowledge of low back pain

The results demonstrated that more than half, 114 (55.6%), of the participants received information regarding their LBP from various sources, including medical officers, physiotherapists, books, Internet, media and schools. The predominant sources of information were medical officers, 38 (33.3%), and physiotherapists, 35 (30.7%). The information obtained was on self-care and the importance of exercises. Less than 1% received information only on contributing factors. Overall, 66 (57.9%) of the participants indicated that the information they received was completely understood by them, while 84 (73.7%) acknowledged that the information was useful to manage their LBP. A chi-squire test revealed a significant relationship between knowledge, attitudes and beliefs of the participants (*p* = 0.04).

## Discussion

### Socio demographic characteristics and low back pain status of participants

The majority of the patients with LBP attending hospital physiotherapy outpatient departments in Malawi were middle-aged women, married, living in an urban area and had a primary level of education. Several population-based studies conducted in both Western and African countries reveal that women are more affected by LBP than men (Sikiru & Hanifa 2010). The female dominance in reported LBP could be because of the fact that women are more likely to seek healthcare for their pain than men and that women may have lower pain thresholds than men (Seotanto, Chung & Wong 2006). Almost half of the participants in our study reported living with chronic recurrent LBP.

### Knowledge of low back pain

Patients with LBP tend to seek information regarding their pain from diverse sources. More than 50% of the participants reported having received information regarding their LBP from medical officers and physiotherapists. The information received included self-care and the importance of exercises for their LBP. Less than 1% received information on the contributing factors for LBP. This was also the case in the study by Tavafian et al. (2004). However, Ng’uurah and Frantz (2006) found that the main reasons for patients seeking healthcare are because of their pain experience as well as because of understanding the causes and available remedies for their pain (Ng’uurah & Frantz 2006). It is well known that patients rely on healthcare providers to understand the causes as well as to educate and advise them on possible management of their health problems (Foster et al. 2003). Therefore, Ng’uurah and Frantz (2006) advised that in order to avoid patients’ misconceptions regarding their LBP, the information should be given in a form that patients can easily understand.

It is therefore essential for healthcare providers and other individuals who are involved in managing patients with LBP to identify the needs and the reasons for patients seeking health services (Nasser 2005). In the management of recurrent LBP, patients’ knowledge regarding the source and mechanism of the pain is important in achieving better treatment outcomes (Ng’uurah & Frantz 2006; Tavafian et al. 2004).Our study results demonstrate that the majority of participants were partially knowledgeable on the course and causes of LBP. Ng’uurah and Frantz (2006) in their study conducted in Kenya also concluded that the majority of patients lacked knowledge regarding the causes and contributing factors for LBP. Similarly, Allock et al. (2007) and Mwilila (2008) found that the majority of patients did not understand the cause of their pain, and the main reason for them visiting healthcare providers was to be educated on the cause of their pain and to seek reassurance regarding the diagnosis and the role of the medication prescribed for their problem. Health education regarding patient’s LBP should be included in the management programme of LBP. Tugwell et al. (2007) proposed that although patients are the experts of their experience of their own illness, they still need to be educated about their illness and its possible causes to enable them to make their own decisions regarding their health. Education has long been used in order to alleviate pain and reduce disability associated with LBP (Udermann et al. 2004). Many studies have examined the effect of education on pain and disability and reported excellent outcomes. The results show that the introduction of an individualised educational booklet on back biomechanics may result in decreased pain and frequency of LBP episodes in patients living with chronic LBP (Coudeyre et al. 2007; Dupeyron et al. 2011; Udermann et al. 2004).

Recent studies evaluated the use of neuroscience education in decreasing pain and disability among patients with LBP (Louw, Nijs & Puentedura 2017). Neuroscience education focuses on neurophysiology and the processing of pain (Louw et al. 2017). Studies that utilise neuroscience education have been shown to decrease fear and change patients’ perception of their pain (Meeus et al. 2010; Ryan et al. 2010). Providing patients with education on pain physiology assists in reconceptualising the concept of pain, enhances the patient’s understanding of their chronic pain and limits the development of inappropriate pain cognitions and negative beliefs (Meeus et al. 2010). Furthermore, it decreases the fear of re-injury among patients, thus enhancing their physical performances (Ryan et al. 2010). A systematic review provided strong evidence for education on pain neuroscience, addressing pain, disability and physical performance in musculoskeletal pain, particularly spinal disorders (Louw et al. 2011). It is therefore clear that the available literature indicates that pain neurophysiology education should be included as part of the management of LBP.

### Attitudes and beliefs regarding low back pain

Ascertaining the attitudes and beliefs patients may hold regarding their pain could facilitate the management of their pain (May 2007). The continuous fostering of negative attitudes and beliefs among patients living with LBP may hinder the achievement of the desired treatment outcomes (Symonds et al. 1996). This implies that changing attitudes and beliefs through education regarding the source and contributing factors to patients’ pain could speed up recovery and enhance earlier return to functional activities (May 2007). The majority of participants in our study indicated negative attitudes and beliefs regarding their own LBP. Participants reported fear of movements and activity avoidance. Three-quarters of the participants believed that their LBP would eventually prevent them from working and that it would remain for the rest of their lives. Darlow et al. (2014) found that the majority of patients believed that movements and physical activity could cause more harm to their LBP, resulting in avoiding certain activities. Hanney, Kolber and Beekhuizein (2008) and Linton, Vlaeyen and Ostelo (2002) indicated that negative beliefs among patients living with LBP may aggravate their pain, leading to functional limitations and chronic pain patterns. It is therefore important, during management of LBP, that patients’ misconceptions regarding their pain be identified and addressed (Darlow et al. 2014).

LBP could occur as a result of many contributing factors and the debate in the literature regarding the exact causes or contributing factors to the occurrence of LBP is still inconclusive. However, both physical and psychosocial factors have been indicated as contributing factors to LBP (George et al. 2006; Heymans et al. 2010; Soucy et al. 2006). The majority of our participants strongly believed that all nine factors (identified in the Delphi study) could contribute to the development or maintenance of their LBP. Our participants strongly believed that factors including repetitive heavy lifting, physically demanding jobs, frequent twisting and bending of the spine and flexion combined with compressive forces to the lumbar spine, fear avoidance beliefs, injury to the back and previous history of LBP could contribute to the occurrence of LBP. Samad et al. (2010) found that factors such as repetitive heavy lifting, prolonged sitting and prolonged flexing of the spine were also indicated as potentially contributing to the occurrence of LBP. Fear avoidance beliefs and somatisation (feeling sick without an actual disease) may also increase the risk of pain chronicity, disability and abstinence from physical activities (George et al. 2006). Because the causes or contributing factors of LBP are several and seldom caused by a single factor (Adam 2009), patients may hold different perceptions regarding the causes of their LBP (Sarah 2000). Therefore, it is important for healthcare providers to identify patients’ perceptions regarding the causes of their LBP because this could help to clear the misconceptions they may hold regarding their pain and could also positively influence their choice of taking up a particular type of treatment (Darlow et al. 2014; Linton, Helsing & Halden 1998).

### Relationship between knowledge, attitudes and beliefs

A statistically significant relationship between knowledge, attitudes and beliefs was confirmed in our study. The knowledge, attitude and beliefs of patients regarding illness and pain are interrelated and inextricable (Bradley 1995). The author further alluded that it is difficult to make a distinction between knowledge and beliefs of the patient (Bradley 1995). Furthermore, Furinghetti and Pehkonen (2002) indicated that a belief is a prerequisite for knowledge and that there is a fine line between belief and knowledge. This implies that the knowledge of patients regarding their condition is linked with the beliefs they hold and their knowledge could influence these beliefs of their pain experience (Gbiri, Olawale & Obi 2015).

## Conclusion

This study highlighted that many patients with LBP in Malawi are not adequately knowledgeable about LBP and hold negative attitudes and beliefs regarding their LBP. Even though no differentiation was made between the knowledge, attitude and beliefs of patients living with chronic versus recurrent LBP, it can be concluded that providing education to the patients regarding their LBP and especially pain neuroscience education may enhance their knowledge regarding LBP. Therefore, LBP management approaches in Malawi should include education programmes aimed at empowering patients with knowledge regarding LBP, its contributing factors as well as changing their negative attitudes and beliefs about their pain. Patients’ understanding of the cause and nature of their pain may enhance the achievement of treatment goals.
